# Reconciling founder variant multiplicity of HIV-1 infection with the rate of CD4^+^ decline

**DOI:** 10.1098/rsif.2024.0255

**Published:** 2024-10-30

**Authors:** James Baxter, Ch. Julián Villabona-Arenas, Robin N. Thompson, Stéphane Hué, Roland R. Regoes, Roger D. Kouyos, Huldrych F. Günthard, Jan Albert, Andrew Leigh Brown, Katherine E. Atkins

**Affiliations:** ^1^ Usher Institute, Edinburgh Medical School, The University of Edinburgh, Edinburgh, UK; ^2^ Faculty of Epidemiology and Population Health, Department of Infectious Disease Epidemiology, London School of Hygiene and Tropical Medicine, London, UK; ^3^ Centre for Mathematical Modelling of Infectious Diseases, London School of Hygiene and Tropical Medicine, London, UK; ^4^ Mathematical Institute, University of Oxford, Oxford OX2 6GG, UK; ^5^ Department of Environmental Systems Science, Institute of Integrative Biology, ETH Zurich, Zurich, Switzerland; ^6^ Department of Infectious Diseases and Hospital Epidemiology, University Hospital Zurich, Zurich, Switzerland; ^7^ Institute of Medical Virology, University of Zurich, Zurich, Switzerland; ^8^ Department of Microbiology, Tumor and Cell Biology, Karolinska Institute, Stockholm, Sweden; ^9^ Department of Clinical Microbiology, Karolinska University Hospital, Stockholm, Sweden; ^10^ Institute of Evolutionary Ecology, The University of Edinburgh, Edinburgh, UK

**Keywords:** infectious disease dynamics, bioinformatics, HIV/AIDS

## Abstract

HIV-1 transmission precipitates a stringent genetic bottleneck, with 75% of new infections initiated by a single genetic variant. Where multiple variants initiate infection, recipient set point viral load (SpVL) and the rate of CD4^+^ T cell decline may be elevated, but these findings remain inconsistent. Here, we summarised the evidence for this phenomenon, then tested whether previous studies possessed sufficient statistical power to reliably identify a true effect of multiple variant infection leading to higher SpVL. Next, we combined models of HIV-1 transmission, heritability and disease progression to understand whether available data suggest a faster CD4^+^ T cell decline would be expected to associated with multiple variant infection, without an explicit dependency between the two. First, we found that most studies had insufficient power to identify a true significant difference, prompting an explanation for previous inconsistencies. Next, our model framework revealed we would not expect to observe a positive association between multiple variant infections and faster CD4^+^ T cell decline, in the absence of an explicit dependency. Consequently, while empirical evidence may be consistent with a positive association between multiple variant infection and faster CD4^+^ T cell decline, further investigation is required to establish a causal basis.

## 1. Introduction

Almost all people living with HIV-1 will progress to AIDS in the absence of treatment, and ultimately die of AIDS-related conditions. The progression rate to AIDS, however, varies considerably between individuals [1,2]. Factors understood to determine the rate of disease progression include characteristics of the infecting virus such as replicative capacity, immunogenicity or pre-adaptation [3–5]; and characteristics of the person such as age and human leukocyte antigen (HLA) phenotype [6–8].

In some viral infections, a worse clinical prognosis has been associated with an increased inoculum size, often described as a ‘dose–response’ relationship [9,10]. For HIV-1, infections initiated by multiple genetically distinct viral variants are associated with elevated set point viral loads (SpVL) and faster CD4^+^ T cell decline [11–15], which are indicative of a faster progression to AIDS when left untreated [3]. Despite 25% of new infections being founded by multiple genetic variants [16–19], a mechanistic understanding of this dose–response relationship remains elusive [15,20].

Recipient SpVL is in part explained by viral genotype, which is inherited from the transmitting individual’s infection. As such, transmitting partners with high SpVLs may be associated with recipient infections with high SpVLs [21–25]. Concomitantly, the probability of a recipient infection being initiated with multiple variants is greater for an individual with higher SpVL, given identical viral diversity [26]. We might expect, therefore, that we should observe individuals with multiple variant infections to have a higher SpVL because their infection would have been more likely initiated by a ‘high SpVL’ genotype. Nonetheless, high viral loads shorten the duration of infection, thereby reducing the likelihood of the higher SpVL individual transmitting and consequently restricting the accumulation of viral diversity. The impact of SpVL on the dynamics of the transmitter’s infection therefore likely determines the need for a causal mechanism that links multiple variants to increased SpVL.

In this study, we test this assumption by first summarising the existing evidence for an association between infections initiated by multiple variants and faster CD4^+^ T cell decline. We then extend a statistical model to evaluate how likely a true difference in progression to AIDS between individuals with and without infections initiated by multiple variants is to be detected by these observational studies. Finally, we construct a model framework that combines a mechanistic transmission model with statistical models to test the hypothesis that we should expect to observe an association between multiple variant infection and prognosis in the absence of an explicit dependency linking the two.

## 2. Methods

### 2.1. Literature review

We first conducted a review to understand the extent to which a difference in HIV-1 prognosis between infections initiated by single and multiple variants is supported. To systematically collate these data, we searched the PUBMED database with the following search criteria: (‘HIV’[Title] OR ‘human immunodeficiency virus type 1’[Title]) AND (‘founder*‘[Title] OR ‘multiplicity’[Title] OR ‘variant*‘[Title]) AND (‘viral loads’[Title/Abstract] OR ‘CD4’[Title/Abstract] OR ‘disease progression’[Title/Abstract] OR ‘poisson’[Title/Abstract]). We restricted our search to studies published between 1 January 2000 and 1 September 2023. Briefly, our search strategy required studies to have reported original estimates of founder variant multiplicity in people with acute or early HIV-1 infections; the recipient partner should not be receiving pre-exposure prophylaxis, and the transmitting partner should be antiretroviral treatment naive. We did not place restrictions on study design, geographical location or on the age of participants. We identified a subset of studies that satisfied our search criteria, which also conducted statistical analyses comparing clinical biomarkers of disease progression between single and multiple variant infections of people living with HIV-1. We recorded the number of study participants, the location of the study, the proportion of the study whose infection was initiated by multiple variants, the method of founder variant quantification, the sampling strategy of viral load and CD4^+^ measurements, and details of the statistical analysis conducted.

### 2.2. Probability of observing a significant association between SpVL and multiple variant infection

We extended a previously described statistical model that illustrates how the distribution of HIV-1 SpVL changes across a transmission cycle [27]. In the original statistical model, the population is subdivided into three groups: carriers, representing people living with HIV, regardless of whether they will go on to be transmitters; transmitters, representing carriers that have transmitted infection to at least one other person; and recipients, representing individuals with recently initiated infections from the transmitters. Making the simplifying assumption that the population of log_10_ SpVL in each group follows a normal distribution, the change in mean and variance of each may be expressed as a series of linear equations. We parameterised the carrier population with mean 4.74 log_10_ copies ml^−1^ and variance 0.78 [27,28].

We then extended this statistical model to consider the findings that infections initiated by multiple founder variants are associated with significantly higher SpVLs than those initiated by single variants. We assume that the normal distribution of recipient SpVLs, X , calculated by Bonhoeffer *et al*.’s model, with mean, μ and variance, $\sigma^{2}$ , can be approximated by a mixture distribution comprising two normal distributions (electronic supplementary material, pages S2 and S3 and figures S1–S3, pages S6–S8). These respective component distributions describe the population of SpVLs attributable to multiple and single variant infections in the transmitter population, contributing to the mixture according to their frequency, p and (1-p) , respectively. The mixture distribution is parameterised such that [29]


(2.1) $$E\left[X\right]\;=\mu \;=p\;\mu_{MV}+\left(1-p\right)\mu_{SV},$$

(2.2) $$E\left[{\left(X-\mu \right)}^{2}\right]\;=\sigma^{2}=p\left(\;\sigma_{MV}^{2}+\mu_{MV}^{2}\right)+\left(\;1-p\right)\left(\;\sigma_{SV}^{2}+\mu_{SV}^{2}\right)-\mu^{2}.$$

Where $\sigma_{MV}^{2}=\sigma_{SV}^{2}$ [13], and with a known effect size representing the true difference in SpVL, $\mu_{MV}$ − $\mu_{SV}$ and known values of μ and p , the component distributions may be parameterised. We inferred component distribution parameters for all combinations of effect size between +0.01 and 1 log_10_ increase SpVL, and values of *p*, between 0 and 0.6. We sampled from the component distributions proportionally to the probability that infection was initiated by multiple variants, *p*, and inferred the significance of the difference between the sample means using a one-sided *t*‐test. We sampled 100 independent draws and recorded the proportion of samples with a significant difference between single and multiple variant log_10_ SpVL distributions.

### 2.3. A model framework integrating transmission dynamics, transmitter and recipient viral loads and CD4^+^ T cell decline

We devised a model framework combining three well-characterised aspects of HIV-1 transmission to identify whether available data on HIV-1 transmission can explain observed associations between multiple variant infection and log_10_ SpVL/CD4^+^ T cell decline. This framework comprises a mechanistic model of HIV transmission, and statistical models of heritability and disease progression. Importantly, this framework assumes no causal relationship between multiple variant infection, and higher log_10_ SpVL or the rate CD4^+^ T cell decline.

### 2.4. Heritability model

Heritability denotes the proportion of variation in SpVL between epidemiologically linked individuals that can be explained by genetic variation of the transmitted virus [21,23]. To predict log_10_ SpVL, y , for individual j in pair i , we fitted a Bayesian linear mixed model to SpVLs of 196 previously determined transmission pairs from the Swiss HIV cohort study (SHCS) [30,31]. SpVL was defined as the geometric mean of viral loads over a period of at least 180 days, obtained prior to commencing antiretroviral treatment and excluding the primary (90 days post-estimated date of infection) and late (CD4^+^ T cell count was below 100 µl^−1^) phases of the infection. For each pair, we fitted a random intercept, $\gamma_{0i}$ , for the within-pair mean log_10_ SpVL and adjusted for key covariates: recipient sex (male, female), age at infection (categorised to intervals of: 16–24, 25–29, 30−39−40−80), partner (transmitter/recipient) and risk group (male–female, female–male and men-who-have-sex-with-men (MSM)).

As the direction of transmission is not known for these pairs, for the purpose of our modelling scenario, we imputed an epidemiological role (transmitter or recipient) for each individual. First, we calculated a theoretical distribution of transmitter log_10_ SpVLs in a population, using a statistical model described by Bonhoeffer *et al*. [27]. We then assigned the role of transmitter within each pair to the individual whose log_10_ SpVL had the highest likelihood of being a transmitter. We conducted two sensitivity analyses on the allocation of transmitter: (i) refitting the heritability model with randomly assigned transmitter status within a transmission pair; and (ii) assigning the individual with highest viral load as the transmitter (electronic supplementary material, pages S4 and S5 and figure S4, page S9)


(2.3) $$\begin{array}{ll} y_{ij\;}= & \;\beta_{0}+\beta_{1}sex_{ij}\;+\beta_{2}age_{ij}+\beta_{3}partner_{ij}\;+\;\beta_{4}riskgroup_{i}+\gamma_{0i\;}+\epsilon_{ij}\\ & \gamma_{0i}∼N\left(0,\sigma_{\mu }^{2}\;\right);\;\;\epsilon_{ij}∼N\left(0,\sigma_{\epsilon }^{2}\right) \end{array}.$$

Priors for the parameter values were informed by Hollingsworth *et al*. (electronic supplementary material, table S1, page S10) [21]. The model was fitted using Markov chain Monte Carlo (MCMC) simulation for 10 000 iterations across four chains. The first 1000 iterations of each chain were discarded as burn-in. Convergence of the joint posterior distribution and adequate mixing of all chains was assessed against criteria of effective sample size, ESS > 1000, and rank-normalised Gelman–Rubin statistic, $\hat{R}$ < 1.05 [32] (electronic supplementary material, table S2 and figure S5, pages S11 and S12). Assumptions of normality and homogeneity of variance were satisfied following visual inspection of QQ and residual plots (electronic supplementary material, figure S6, page S13). Predicted values of heritability were drawn from the posterior predictive distribution, calculated as the variance of the predicted SpVL divided by the sum of the variance of predicted SpVL and the expected variance of the errors (electronic supplementary material, figure S7, page S14) [33].

### 2.5. Tolerance model

We used a previously described statistical model that characterises the relationship between recipient SpVL and CD4^+^ T cell decline [34,35]. In brief, this model was fitted to SpVLs and longitudinal CD4^+^ T cell counts of 3036 people living with HIV-1 who participated in the SHCS. In their analysis, Regoes *et al*. [34] found that the rate of change of CD4^+^ T cells per ml of blood per day, *y*, for recipient, *j*, was best expressed as a quadratic function of SpVL, $\log_{10}V_{j}$ . The tolerance at birth for females is given by, $\;\alpha_{\left(0,F\right)},\;$ which thereafter changes linearly with age, a , at rate $c_{F}$ , per year. The sex difference between tolerance at birth, $\eta_{\left(0,M\right)}$ , and the sex difference between the change of tolerance per life year, $M_{\left(0,M\right)}$ , represent the linear effects of recipient age and sex, and their interaction,


(2.4) $$y_{j}=\left[\alpha_{0,F}+\eta_{0,M}+\left(c_{F}+z_{M}\right)a\right]{\left(\log_{10}V_{j}\right)}^{2}.$$

During model fitting, Regoes *et al*. excluded the linear viral load term and the intercept, finding that they did not deviate significantly from zero. The parameter values used in this study are detailed in the electronic supplementary material, table S3, page S15.

### 2.6. Transmission model

We extended a previously described mechanistic transmission model that links transmitter infection viral load with the probability of recipient infection being initiated by multiple viral variants [26]. The model calculates the number of virus particles, *n*, and number of viral variants, *N*, initiating infection in the recipient partner at a given time τ during the transmitter partner’s infection. The number of virus particles founding infection at transmission time, τ , is distributed binomially, n(τ)~Bin(ν(τ),p) , assuming an inoculum of size, ν(τ) , which is proportional to viral load at transmission and a per-particle establishment probability, *p*. The marginal probability that a given number of variants initiates infection, P(N(τ)) , is given by $P\left(N\left(\tau \right)\right)\;∼\;\sum_{n\left(\tau \right)=N\left(\tau \right)}^{v}P\left(N\left(\tau \right)|n\left(\tau \right)\right)$ . As in the original model, we reconciled the low probability of infection acquisition with a relatively high proportion of multiple variant infections, by assuming only a fraction, *f*, of exposures occur in an environment conducive to transmission resulting in a zero-inflated probability distribution.

The number and diversity of virus particles are modelled across the duration of the transmitter infection. Similar to Thompson *et al*. [26], we assumed that no transmission occurs during AIDS due to the severity of illness and categorised the remaining infectious period into primary, chronic and pre-AIDS. We then extended the model to relax the previous assumption of fixed viral loads in primary and pre-AIDS infection (electronic supplementary material, figure S8A, page S16). Specifically, we assumed that viral load varied by Fiebig stage during primary infection, with stages of length 5 days (stage I), 5.3 days (stage II), 3.2 days (stage III), 5.6 days (stage IV) and 69.5 days (stage V) [36]. Within each stage, we assumed the log_10_ viral load profiles follow a normal distribution, calculated the probability that infection was initiated by multiple variants for the 0.1, 0.15, 0.5, 0.85, 0.9 percentile viral load and weighted by their respective probability density (electronic supplementary material, figure S8B, page S16). For pre-AIDS infection we fitted a lognormal distribution to previously described viral loads using maximum likelihood, then calculated the probability that infection was initiated by multiple variants at discrete intervals, weighted according to their respective probability density [37]. The best-fit distribution for pre-AIDS viral loads was parameterised by mean $\log_{10}1.62$ and standard deviation $\log_{10}0.114$ (electronic supplementary material, figure S8C, page S16). Finally, we followed Thompson *et al*. in assuming that a small number of variants predominate early infection, giving way to a more uniform distribution of variants (synonymous with higher diversity) as infection progresses (electronic supplementary material, figure S8E, page S16) [38]. We modelled the proportion of the xth most common viral variant at time, τ , since infection, as a gamma distributed random variable, X~Γ(j,k) , with shape parameter, j=0.417 , and scale parameter, k=τ/0.563 . All other fixed parameters remained the same as first presented by Thompson *et al*. (electronic supplementary material, table S4, page S15).

To calculate the probability that an infection is initiated by multiple variants for a given transmitter SpVL, we first estimated risk group stratified parameter values *f* and *p,* integrated across all infected potential transmitters in the population, and all times during their courses of infection. Whereas Thompson *et al*. [26] set the per particle transmission probability, *p*, and the proportion of the time the recipient environment is conducive for transmission, *f*, such that the conditional probability of transmitting multiple variants in a single act was 0.3, we used a Bayesian model fitting approach to infer joint posterior distributions of *f* and *p* for a given per-event probability of acquisition and the observed probability that infection is initiated by multiple founder variants (electronic supplementary material, figure S9, page S17). We ran two independent MCMC simulations for 30 000 iterations with a 25% burn-in for each risk group. After concatenating the two chains, a minimum ESS of 2000 was achieved across all analyses, with a Gelman–Rubin statistic of $\hat{R}$ < 1.05. Joint posterior distributions of *f* and *p* recapitulated empirical distributions of the per-event probability of acquisition and the probability that infection is initiated by multiple founder variants (electronic supplementary material, figure S10, page S18) [19,39]. Parameter values of *f* and *p* were drawn from the joint posterior distribution and used to calculate individual probabilities that an infection is initiated by multiple variants, for a given transmitter SpVLs integrated over the course of that transmitter’s infection.

### 2.7. Risk group-stratified cohorts

To disentangle variation associated with sex and age across risk groups, we simulated risk group-stratified data from the 196 SHCS transmission pairs used in our heritability analysis. First, we selected only those pairs whose risk groups were reversible and calculated separate variance–covariance matrices for each risk group of mean log_10_ SpVL, age at infection and sex pairs. Next, we used these relationships to synthesise populations of size 200, from a truncated multivariate normal distribution. Categorical variables were enumerated prior to simulation and reclassified according to the cumulative probability distribution calculated for each variable from the empirical data. We truncated ‘age at infection’ with a lower bound of 16. We validated these results by comparing the difference in the means and variance between the stratified simulated and empirical data (electronic supplementary material, figure S11, page S19).

## 3. Computation

All analyses were implemented in R v. 4.1.2 [40]. The heritability model was fitted in Stan using BRMS v. 2.20.4 [41,42]. Extensive use was made of the Tidyverse suite v. 2.0.0 for data handling; and tidybayes v. 3.0.6, emmeans v. 1.8.9 , bayesplot v. 1.10.0 and performance v. 0.10.5 for post-processing [43–46].

## 4. Results

### 4.1. Observational evidence for an association between infections initiated by multiple variants and faster CD4^+^ T cell decline

We conducted a systematic search of the PUBMED database, which returned 292 unique studies. We identified five studies that characterised associations between founder variant multiplicity, viral load and CD4^+^ T cell decline across six epidemiological cohorts (table 1) [11–15]. All studies inferred a binary ‘single/multiple’ classification of founder variant multiplicity, and all studies modelled the outcome of multiple variant infection on viral load. Five of the six epidemiological cohorts were analysed for a CD4^+^ T cell outcome. Heterosexual transmissions were analysed by Sagar *et al*. [11], Abrahams *et al*. [12] and Janes *et al*. [13], while MSM transmissions were also analysed in Janes *et al*., Chaillon *et al*. [14] and Macharia *et al*. [15].

**Table 1.** Summary of the cohort characteristics and key results from studies that have analysed the association between CD4^+^ T cell decline and infections initiated by multiple founder variants. Risk groups: HSX, heterosexual; MSM, men-who-have-sex-with-men; MF, male-to-female. Probability of multiple variants, *p*, as estimated by the original studies using the methods described. Study design: RCT, randomised controlled trial. Methods: heteroduplex assays distinguish genetically similar and dissimilar genomic segments using gel electrophoresis of viral RNA; mathematical model and sequence diversity/genetic distance methods test whether the observed diversity fits an assumed threshold or distribution of diversity that would be expected under a hypothesis of neutral exponential growth from a single variant; visual haplotype methods may be used to identify clusters of sequences sharing polymorphisms. Recipient phylogenetic methods test whether the phylogeny estimated from the recipient sequences is consistent with the star-like topology expected for a single variant infection. Phylogenies that use both source and recipient sequences from known transmission pairs allow us to estimate the number of founder variants by counting the number of source phyly nested within the source sequences. Sampling: EDI, estimated date of infection. Analysis: MW test—Mann–Whitney *U*-test; LMM, linear mixed model. Effect size: Sqrt CD4, square root CD4^+^ T cell count. Significance: ns, *p* > 0.05; *p ≤ 0.05; **p ≤ 0.01; ***p ≤ 0.001 as measured in the original studies.

	number of individuals	location	risk group	study type	multiple variant detected (%)	method of variant multiplicity detection	sampling details	analysis	effect size (with significance)	analysis	effect size (with significance)
[11]	156	Kenya	HSX (MF)	prospective serodiscordant couples	57	heteroduplex mobility assay	median 6 samples (range: 1–26) over median 907 (range: 14–2933) days. Median time to first sample: 75 days	MW test for primary infection, LMM for longitudinal analysis	+0.27*	MW test for primary infection; Kaplan–Meier curves and log-rank test for longitudinal analysis	*
[12]	90	South Africa; Malawi	HSX (MF)	prospective serodiscordant couples (*n* = 69); observational clinic study acute infections (*n* = 21).	22	distribution of hamming distances, sequence alignments, recipient phylogenetic topology, mathematical model	24 individuals with absolute viral load and CD4^+^ counts at 12 months post-infection	undescribed univariate test of difference between means	— (ns)	undescribed univariate test of difference between means; Fisher’s exact test to compare founder multiplicity and fast/slow progressor status	— (ns)
[13]	100	Thailand (RV144)	HSX	vaccine RCT	32	sequence alignments, recipient phylogenetic topology and sequence diversity	initial plasma samples at diagnosis, then 1, 3, 6, 9, 12, 18 and 24 months	weighted generalised estimating equations	+0.239**	weighted generalised estimating equations	−2.112 Sqrt CD4*
	63	Canada; Peru; USA (STEP)	MSM	vaccine RCT	25		initial plasma samples at diagnosis, then at 1, 2, 8, 12, 26, 52, 78 and 104 weeks		+0.372***		−0.507 Sqrt CD4 (ns)
[14]	26	USA	MSM	case series	53	genetic distance, source/recipient phylogeny topologies	samples collected mean 70 days (range 11−170) after EDI	undescribed univariate test of difference between means	—	NA	NA
[15]	38	Kenya	MSM	prospective cohort study	39	distribution of hamming distances, sequence alignments, recipient phylogenetic topology.	first sample at 21 (range:12–59) days post-infection. 27 patients on monthly follow-up and 10 on quarterly follow-up for 5 years	MW test to compare SpVL	—(ns)	Kaplan–Meier curves and log-rank test	—***

Multiple variant infection was associated with significantly higher viral load at diagnosis in four epidemiological cohorts. To compare viral loads between infections initiated by single and multiple variants, Abrahams *et al*., Chaillon *et al*. and Macharia *et al*., applied univariate statistical tests. Sagar *et al*. and Janes *et al*. applied linear mixed-effects models and generalised linear models, respectively. Janes *et al*. included key covariates, such as sex, age and human leukocyte antigen genotype, whereas all other studies reported unadjusted estimates. Effect sizes were reported for three epidemiological cohorts and were broadly consistent (table 1). For the studies that analysed repeat viral load measurements, the presence of a significant difference between single and multiple variant infections varied throughout infection. For example, at the time of diagnosis, higher viral load was not associated with multiple variant infection in Janes-STEP, but at each time-point thereafter, higher viral load was associated with multiple variant infection. In both epidemiological cohorts analysed by Janes *et al.*, the magnitude of the increase in viral load due to multiple variant infection decreased over time.

Of the four epidemiological cohorts in which a significant association between viral load and multiple variant infection was identified, a further association with lower CD4^+^ T cell count or faster CD4^+^ T cell decline was established in two. Mean square root CD4^+^ T cell counts over the first year of infection were analysed by Janes *et al*., revealing a significant decrease in CD4^+^ T cell count associated with multiple variant infection in Janes-RV144. Meanwhile, Sagar *et al*. had performed a survival analysis of the proportion of individuals with a CD4^+^ T Cell count above 350 cells mm^−3^, stratified by founder variant multiplicity. This analysis revealed women infected with multiple variant infections would reach a CD4^+^ T cell count of less than 350 cells mm^−3^ in 39.2 months (95% confidence interval (CI): 30.8–47.6), significantly faster than 56.8 months (46.0–67.7) for those with single variant infections. A second study, Macharia *et al.*, also identified a significantly greater rate of decline of the proportion of individuals with a CD4^+^ T Cell count above 350 cells mm^−3^, but did not also identify an association with SpVL. One cohort did not demonstrate any association between multiple variant infection and either viral load or CD4^+^ T cell decline [12].

### 4.2. Do we expect to observe an association between multiple variant infection and log_10_ SpVL in an observational study?

Previous observations linking multiple variant infection to a higher SpVL and faster rate of CD4^+^ T cell decline are inconsistent. To determine whether such studies had sufficient statistical power, we tested how likely it is that a true difference in SpVL between individuals with and without infections initiated by multiple variants would have been detected. To compare the probability of observing a significant difference between the log_10_ SpVL of infections initiated by single and multiple variants, we first modelled changes in the distribution of log_10_ SpVL over a transmission cycle (see electronic supplementary material, pages S2 and S3) [27]. Extending this statistical model, we approximated the recipient log_10_ SpVL distribution with two normal distributions of equal variance, representing single and multiple variant infections. The difference in the means was assumed to be the signed effect size of observational studies (table 1). We then sampled the distributions of single and multiple variant infection log_10_ SpVL, each proportional to the frequency of single and multiple variant infections in the population*,* and tested if the samples were significantly different.

**Figure 1.**
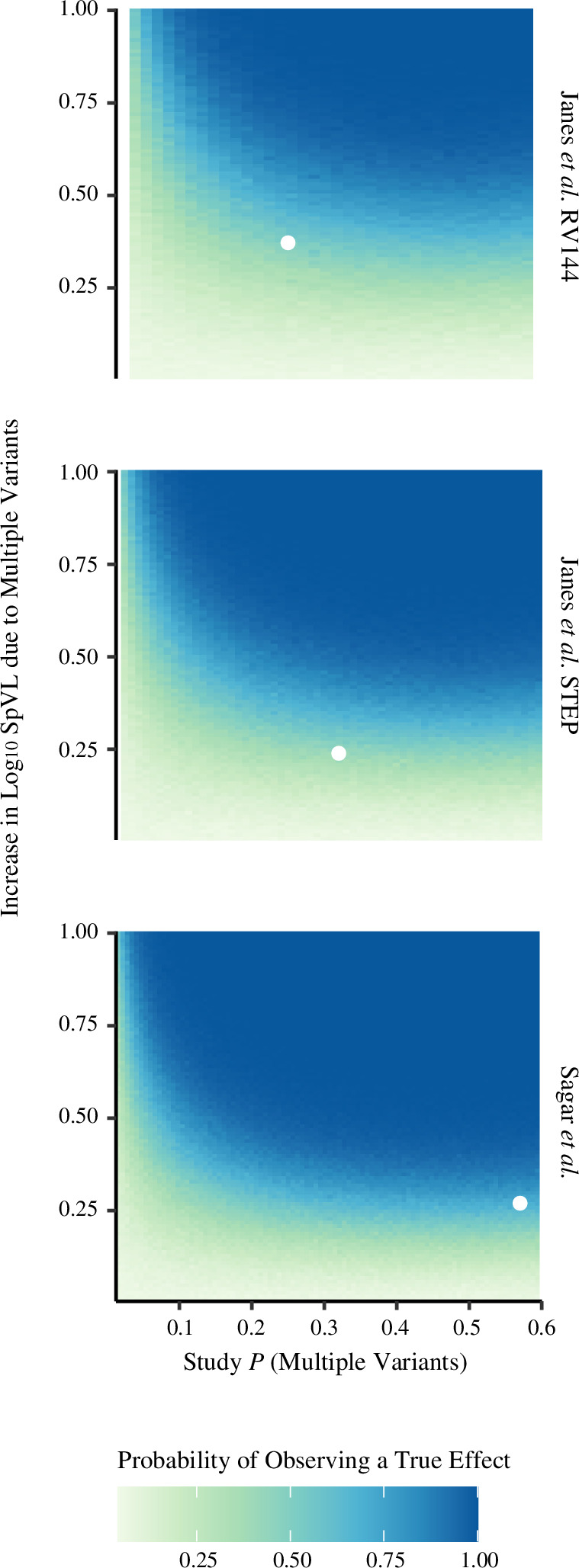
For each study that calculated an effect size, we estimated the power of each study to detect a significant difference in SpVL between individuals with and without infections initiated by multiple founder variants. Scaling with study size (*n*), an effect size threshold is apparent, above which one is more likely than not to observe a significant difference in SpVL between single and multiple variant infections. The probability that infection is initiated by multiple variants greatly increases the probability of observing a true difference in SpVL due to founder variant multiplicity, saturating between 0.2 and 0.3, depending on study size. The effect sizes calculated by each study and the frequency of multiple variant infections are shown as white-filled circles.

Across different study sizes, we found that the probability that a true difference in log_10_ SpVL between individuals with and without infections initiated by multiple variants will be detected by an observational study varies considerably (figure 1). Given the signed effect sizes of multiple variant infection on log_10_ SpVL by Janes *et al*. and Sagar *et al.*, and their respective study sizes, we calculated the proportion of observational studies likely to detect a true significant effect of multiple variant infection. For Janes-RV144, we estimated that one would expect to record a significant difference in log_10_ SpVL between single and multiple SpVL in less than half (0.411 (95% CI: 0.381–0.441)) of cohorts. Conversely, for Janes-STEP we estimated that one would be equally as likely to record a significant difference than not (0.517 (0.486–0.548)), and for Sagar *et al*. that one would be more likely than not to record a significant difference (0.672 (0.643–0.701)).

Our analysis also shows that for given effect size, the probability that infection is initiated by multiple variants influences the probability of observing a true difference between multiple and single variant SpVLs. The probability that infection is initiated by multiple variants differs significantly by risk group [19], therefore analysing one specific risk group could determine how likely one is to record a significant association between multiple variant infection and greater log_10_ SpVL. To illustrate this, we assume a cohort of size 66 and an effect size of 0.27 log_10_ copies ml^−1^ (medians, table 1). The probability of observing a significant difference between the SpVLs of infections initiated by multiple and single variants in a male-to-female cohort is 0.289 (95% CI: 0.262–0.318), compared with 0.257 (0.231–0.285) for a female-to-male infection cohort, and 0.367 (0.338–0.397) for a MSM cohort. Compared with a male-to-female cohort, we are significantly more likely to observe a true difference between the SpVLs of infections initiated by single and multiple in a MSM cohort (odds ratio (OR) 1.61 (95% CI: 1.31–1.97). On the contrary, we are significantly less likely to observe a true difference in a female-to-male cohort (OR 0.562 (0.442–0.713)). Altogether, these results suggest that most observational studies are insufficiently powered and are not certain to consistently detect a true difference in log_10_ SpVL between individuals with and without infections initiated by multiple variants.

### 4.3. Using available data on HIV-1 transmission, our model framework cannot recapitulate the association between multiple variant infection and log_10_ SpVL/CD4^+^ T cell decline

**Figure 2.**
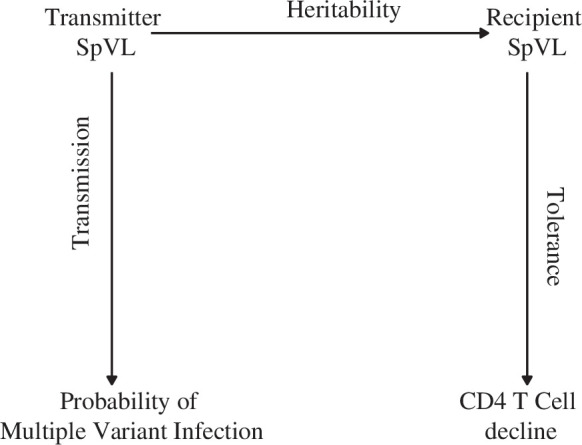
To evaluate the relationship between the probability that infection is initiated by multiple variants, and the rate of CD4^+^ T cell decline, we leverage three well-characterised empirical relationships: we predict recipient SpVL as a function of transmitter SpVL; calculate the probability that a potential recipient infection is initiated by multiple variants, as a function of transmitter SpVL; and predict the daily rate of CD4^+^ T cell decline (ΔCD4) from recipient SpVL.

**Figure 3.**
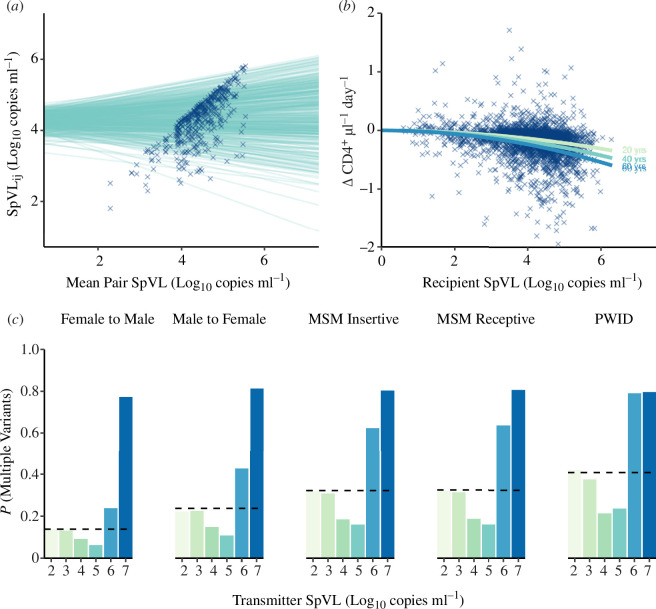
Components of the model framework. (*a*) *Heritability model*: a linear mixed model predicting the SpVL for individual *j* in transmitter–recipient pair *i* as a function of the pair’s mean SpVL. (*b*) *Tolerance model*: a nonlinear model that predicts the rate of CD4^+^ T cell decline for a given recipient SpVL, accounting for linear effects of age and sex. (*c*) *Transmission model*: a mechanistic model to calculate the probability that an infection is initiated by multiple variants, given a transmitter SpVL. We fitted this model for each risk group using previously estimated probabilities of acquisition and that infection was initiated by multiple variants (dashed line).

To establish whether available data can explain the probability that infection is initiated by multiple variants, SpVL and the rate of CD4^+^ T cell decline, we designed a model framework characterising HIV-1 transmission and disease progression (figures 2 and 3). First, we calculated the probability that a recipient’s infection was initiated by multiple variants as a function of the transmission pair’s SpVL (the ‘transmission model’). Second, we calculated the recipient partner’s SpVL as a function of their transmitting partner’s SpVL (the ‘heritability model’) (figure 3*a*). Third, we calculated the rate of a recipient’s CD4 cell decline as a function of their SpVL (the ‘tolerance model’) (figure 3*b*) [34]. Using these models, we were able to infer the extent to which SpVL or CD4^+^ T cell decline varied as a function of the probability that infection was initiated by multiple variants in a risk group stratified population of people living with HIV. Importantly, this model assumes no causal relationship between multiple variant infection, higher log_10_ SpVL or the rate CD4^+^ T cell decline.

The heritability and transmission models were newly fitted for this study. We estimated the broad sense heritability, $H^{2},$ of log_10_ SpVL using a Bayesian linear mixed model and calculated an approximate *R* ^2^ value. For this study, we assumed heritability measures the extent to which genetic variation between infecting viral lineages explains the observed variation of log_10_ SpVL. Including a random intercept for each pair, and adjusting for recipient sex, age at infection, partner and risk group, we estimated the $H^{2}$ to be 0.238 (95% highest posterior density: 0.123–0.341). When fitting our transmission model, the probability that infection is initiated by multiple variants was lowest at intermediate transmitter SpVLs (P(MV) = 0.14 at 4 log_10_ copies mm^−3^ for male-to-female transmissions), but more likely at higher transmitter SpVLs (P(MV) = 0.81 at 7 log_10_ copies mm^−3^ for male-to-female transmissions) (figure 3*c*). Lower transmitter SpVLs were approximately representative of the risk group average probability that infection was initiated by multiple variants (P(MV) = 0.22 at 2 log_10_ copies mm^−3^ for male-to-female transmissions).

**Figure 4.**
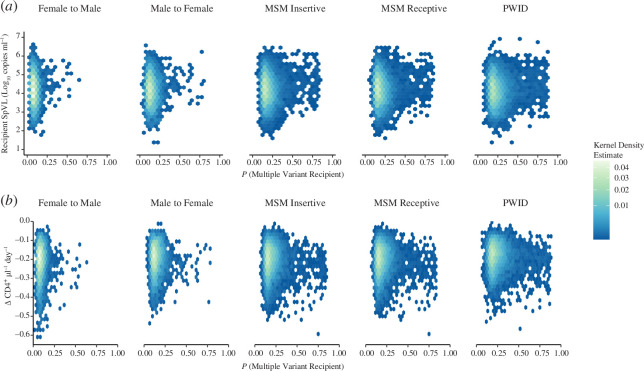
Kernel density estimates of recipient SpVL and the probability that the infection was initiated by multiple variants (*a*), and the rate of decline of CD4^+^ T cells and the probability that infection was initiated by multiple variants (*b*), stratified by risk group.

Applying our model framework to simulated risk group-stratified transmission pairs, we inferred relationships between the probability that infection is initiated by multiple variants, recipient log_10_ SpVL and the rate of CD4^+^ T cell decline. Across risk groups, we found that recipient infections were most likely to have a low probability of being initiated by multiple variants and present with intermediate log_10_ SpVL (figure 4*a*). Specifically, the median probabilities that infection was initiated by multiple variants were 0.173 (interquartile range (IQR): 0.147–0.221) for receptive anal intercourse in men-who-have-sex-with-men (MSM-receptive), 0.212 (0.178–0.276) for people-who-inject-drugs (PWID), 0.131 (0.108–0.167) for male-to-female, 0.080 (0.064–0.104) for female-to-male and 0.174 (0.146–0.222) for insertive anal intercourse in men-who-have-sex-with-men (MSM-insertive). For risk groups associated with a greater risk of multiple variant infection, such as MSM-receptive and PWID, most infections would be expected to have a low probability of being initiated by multiple variants, except a small proportion of infections initiated with multiple variants with very high probability (approx. 0.8). There was no clear trend between these relatively high probabilities that infection is initiated by multiple variants and high recipient log_10_ SpVL.

Across risk groups, we predicted a relatively slow rate of CD4^+^ T cell decline, determined as a function of recipient log_10_ SpVL, age and sex. Specifically, the median rates of daily CD4^+^ T cell decline were −0.200 (IQR: −0.250 to −0.154) for MSM-receptive, −0.177 (−0.225 to −0.138) for PWID, −0.189 (−0.239 to −0.145) for male-to-female, −0.204 (−0.260 to −0.158) for female-to-male and −0.200 (−0.250 to −0.154) for MSM-insertive (figure 4*b*). For risk groups associated with a greater probability of multiple variant infection, such as MSM-receptive and PWID, the overall distribution of the daily rate of CD4^+^ T cell decline remained consistent with risk groups associated with a lower probability of multiple variant infection. Within each risk group, where an infection was predicted to be initiated with multiple variants at a high probability, this did not correspond to a faster rate of CD4^+^ T cell decline. Qualitatively, our findings were consistent across transmitter allocation methods and when log_10_ SpVL and CD4^+^ T cell decline were compared with the probability that infection is initiated by multiple particles (electronic supplementary material, figures S12 and S13, pages S20 and S21).

**Figure 5.**
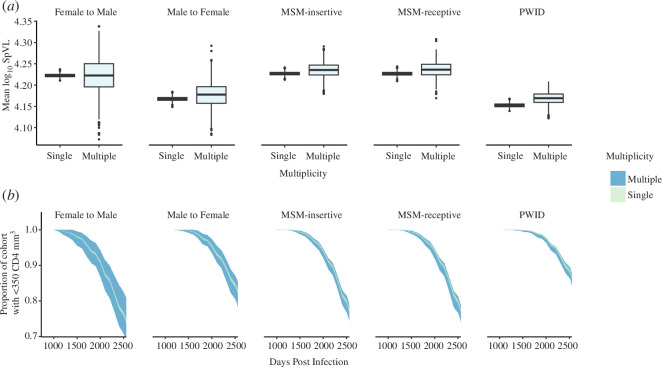
(*a*) Box plots comparing the distribution of the resampled mean SpVLs across transmission groups. (*b*) Extrapolating the proportion of individuals with less than 350 CD4 T cells mm^−3^ from linear rates of decline and resampling according to the probability that infection was initiated by multiple variants. Upper and lower bounds of the ribbons represent the 5th and 95th quantiles.

To compare our model framework with the outcome variables used in observational studies, we calculated the mean SpVLs for single and multiple variant infection as well as the proportion of each risk group that would have an excess of 350 CD4^+^ T cells mm^−3^ over time. The average log_10_ SpVL of female-to-male infections initiated by multiple variants was the same as single variant infections (median 4.22 (IQR: 4.20–4.25) log_10_ copies mm^−3^; 4.22 (4.22–4.23) log_10_ copies mm^−3^, respectively) (figure 5*a*). The average log_10_ SpVLs of male-to-female, MSM-receptive, MSM-insertive and PWID infections initiated by multiple variants were slightly greater than infections initiated by a single variant (4.18 (4.16–4.20) log_10_ copies mm^−3^ and 4.17 (4.16–4.17) log_10_ copies mm^−3^; 4.24 (4.22–4.25) log_10_ copies mm^−3^ and 4.23 (4.22–4.23) log_10_ copies mm^-3^; 4.24 (4.22–4.25) log_10_ copies mm^−3^ and 4.23 (4.22–4.23) log_10_ copies mm^−3^; and 4.17 (4.16–4.18) log_10_ copies mm^−3^ and 4.15 (4.15–4.16) log_10_ copies mm^−3^, respectively). Across all risk groups, log_10_ SpVLs of single variant infection were significantly less dispersed than those from infections initiated by multiple variants (paired, single-tailed *t*‐test, *p* = 0.0027).

We also found that the difference in time to a clinically relevant (≤350 cells mm^−3^) CD4^+^ T cell count between multiple and single variant infections varied slightly across risk groups (figure 5*b*). In MSM-receptive transmissions, the time to a clinically relevant CD4^+^ T cell count for single variant infections was 9.96 years, compared with 9.92 years for infections initiated by multiple variants. In both male-to-female and PWID transmissions, however, the median time to a clinically significant CD4^+^ T cell count in multiple variant infections was the same as that for single variant infections (10.7 and 10.9 years, respectively). In female-to-male and MSM-insertive transmissions, the time to a clinically significant recipient CD4^+^ T cell count for infections initiated by a single variant (9.78, 9.95) was actually slightly shorter than those initiated by multiple variants (9.93, 9.97). Across all risk groups, the median time to a clinically significant recipient CD4^+^ T cell count associated with single variant infection was significantly less dispersed than those from infections initiated by multiple variants (paired, single-tailed *t*‐test, *p* = 0.003).

## 5. Discussion

In this study, we sought to understand whether the significant association between multiple variant infection, log_10_ SpVL or faster CD4^+^ T cell decline could be recapitulated using available data, in the absence of an explicit dependency. First, we identified six epidemiological cohorts in which associations between multiple variant infection, viral load and CD4^+^ T cell decline had previously been investigated. We then showed how most observational studies lacked the statistical power to consistently identify a true effect. Indeed, the probability of observing a true difference in SpVL due to multiple variant infection could depend on study parameters and the risk group analysed. Next, we tested whether, using available data, we would expect to observe the reported association between multiple variant infection and HIV-1 prognosis. Our model framework revealed we should not expect a high probability of multiple variants initiating infection to coincide with high log_10_ SpVL and a faster rate of CD4^+^ T cell decline. Interpreting our probabilistic output through median log_10_ SpVL and the proportion of individuals with greater than 350 CD4^+^ T cells mm^–3^, we found that both end-points were similar between multiple and single variant infections and varied only slightly across risk groups. Both log_10_ SpVL and the time to a clinically significant CD4^+^ T cell count, showed significantly greater variation in multiple variant infections than infections initiated by a single variant across risk groups.

Altogether, our results are consistent with the hypothesis that a causal mechanism is required to recapitulate the observed association between multiple variant infection and HIV-1 prognosis. This suggests that a simple association between higher viral load and greater diversity in the transmitter that leads to higher viral load in the recipient, irrespective of the number of variants initiating infection, is itself an insufficient explanation. Across an infection cycle, individuals with intermediate log_10_ SpVL infections are more likely to pass on their infection than those with higher viral loads [28]. According to our transmission model, infections acquired from such individuals are initiated by multiple variants with low probability while multiple variant infections initiated by individuals with high log_10_ SpVL occur more frequently. Accordingly, to observe an association between high log_10_ SpVL and multiple variant infection without a within-patient mechanism, log_10_ SpVL would need to be predominantly determined by the transmitted genotype. Instead, we calculated the broad sense heritability of log_10_ SpVL to be 23.9%, meaning the log_10_ SpVL of the recipient infection is only weakly determined by the genotypic load of the transmitter virus population.

Presently, a causal link between the number of variants initiating HIV-1 infection and disease prognosis is yet to be identified. Where an infection is initiated by multiple variants, this is associated with a weaker selective transmission bottleneck such that any ‘reasonably fit’ virus may initiate infection [47,48]. Founder viruses isolated from patients whose infection was initiated by multiple variants have shown a worse replicative capacity *in vitro* than those initiated by single variants [15]. This may suggest that it is not an inherent virological property of the founder viruses that drives an association with a faster rate of CD4^+^ T cell decline, but instead how the presence of multiple variants determines disease dynamics in early infection. Multiple variant infections typically possess one major variant and one or more minor variants at a single point in time [15,49,50]. The relative proportions of these variants in major compartments have been shown to vary throughout early infection, in addition to prolific inter-variant recombination [49,51,52]. Nonetheless, key phenotypes such as coreceptor usage efficiency, IFN-α resistance and viral fitness are not associated with the proportions of founder variants in each individual during acute infection [20]. It has been speculated that the shifts in variant proportion in early infection are immune driven, with minor variants cyclically out-competing the dominant variant due to a non-negligible selective advantage [49]. Given that multiple variant infections lead to the earlier presence of broadly neutralising antibodies, it may follow that in trying to mount a sufficiently broad immune response, initial efficacy is attenuated and leads to increased sequestration and higher viral loads during the chronic phase [53,54].

We were unable to account for several contributors to HIV-1 disease progression in our models. The role of human genetic variability, in particular specific HLA genotypes that modulate resistance to infection, is well documented and may have a key role in determining SpVL, in particular when transmitter and recipient are phenotypically similar [8,55]. Crucially, should the interaction between HLA and viral genotype determine log_10_ SpVL in the recipient, independent action assumptions in our model of heritability would be contravened [5]. Viral properties, such as immunogenicity and co-receptor usage, may also contribute to the rate of CD4^+^ T cell decline independent of log_10_ SpVL, but remain unaccounted for [56]. Our model framework also highlights limitations in our understanding of HIV-1 transmission. Our transmission model assumes no mutation, selection and recombination; all of which may influence the within-person fitness of the infecting virus [51,52]. The heritability model links the within-person processes of the transmission model to the tolerance model; mid-parent offspring regression estimates of heritability are infamously low-powered, however, and we cannot exclude the possibility that within phylogenetically linked transmission pairs, infection of a seronegative partner occurred from an unknown third party or a common source [57]. Furthermore, the precise quantification of pathogen trait heritability remains challenging, with estimates of HIV-1 virulence heritability varying between 7% and 40% [21–25,58,59].

In summary, our model framework cannot recapitulate the observed associations between multiple variant infection and faster CD4^+^ T cell decline in the absence of an explicit dependency between the two. Moreover, given the existence of a true effect, our statistical model has shown that most observational studies lack the power to reliably detect it. Ultimately, there remain key shortcomings in our knowledge of events happening both during and just after transmission, and whether these events determine HIV-1 prognosis. This phenomenon would benefit from further study through a causal framework, incorporating transmitter and recipient viral loads, the time since infection sampling and the number of variants initiating infection, should data be available.

## Data Availability

The tolerance and heritability models were fitted to data from the Swiss HIV cohort study (SHCS). These data are too comprehensive to preserve patient privacy in persons living with HIV and will not be made openly available. The SHCS informed consent states that sharing data outside the SHCS network is only permitted for specific studies on HIV infection, and only to researchers who have signed an agreement detailing the use of these data. A data transfer request may be made at https://www.shcs.ch/308-material-and-data-transfer-agreement, subject to the approval of the SHCS Scientific Board. All remaining data and code have been made available [60]. Supplementary material is available online.
